# Three Cases of Congenital Rubella Syndrome in the Postelimination Era — Maryland, Alabama, and Illinois, 2012

**Published:** 2013-03-29

**Authors:** Kurt Seetoo, Maria Paz Carlos, David Blythe, Leena Trivedi, Robert Myers, Tracey England, Criscelia Agee, Bill Arnold, Carolyn Dobbs, Mary McIntyre, Enrique Ramirez, Julie Morita, Saadeh Ewaidah, Wilete Ishow, Teresa Chou, Kenneth Soyemi, Albert E. Barskey, Amy Parker Fiebelkorn, Paul Lucas, Emily S. Abernathy, Joseph P. Icenogle, Gregory S. Wallace, Susan E. Reef, Yoran Grant

**Affiliations:** Maryland Dept of Health and Mental Hygiene; Alabama Dept of Public Health; Chicago Dept of Public Health; Advocate Illinois Masonic Medical Center; Illinois Dept of Public Health; National Center for Immunization and Respiratory Diseases; Center for Global Health; EIS Officer, CDC

Infection with rubella virus during pregnancy, especially during the first trimester, can result in congenital rubella syndrome (CRS). Serious manifestations of CRS include deafness, cataracts, cardiac defects, mental retardation, and death (1). In the last major rubella epidemic in the United States, during 1964–1965, an estimated 12.5 million rubella virus infections resulted in 11,250 therapeutic or spontaneous abortions, 2,100 neonatal deaths, and 20,000 infants born with CRS (2). In 2004, after implementation of a universal vaccination program, elimination of endemic rubella virus transmission was documented in the United States; evidence also suggests that endemic rubella has been eliminated in the entire World Health Organization (WHO) Region of the Americas (3,4). However, rubella virus continues to circulate elsewhere in the world, especially in regions where rubella vaccination programs have not been established (e.g., the African Region), placing the United States at risk for imported cases of rubella and CRS. During 2004–2012, 79 cases of rubella and six cases of CRS were reported in the United States (Figure); all of the cases were import-associated or from unknown sources. Of the three cases of CRS that occurred in 2012, conditions included cardiac defects, cataracts, hearing impairment, and pericardial effusion in one infant; patent ductus arteriosus, cardiomegaly, thrombocytopenia, and pneumonitis in a second infant; and cataracts, thrombocytopenia, and cardiac defects in a third infant. All three mothers had been in Africa early in their pregnancies. While rubella remains endemic elsewhere in the world, imported CRS will continue to be a public health concern in the United States.

## Case Reports

### Infant A

In February 2012, an infant born in Maryland at 36 weeks’ gestation and weighing 4.2 lbs (1,910 g) was noted at birth to have congenital heart defects, hyperpigmented skin lesions, cataracts, cerebral edema, and pericardial effusion. Hearing impairment was suspected after the infant failed a hearing screening test before hospital discharge in February, and bilateral profound hearing impairment was diagnosed by an audiologist in June. Surgical procedures for correction of congenital heart defects and cataracts were performed in February and June, respectively. During eye surgery, the infant experienced breathing difficulties and went into cardiac arrest. Following stabilization, the infant was admitted to the pediatric intensive-care unit for observation and was later discharged.

CRS diagnosis initially was confirmed at a commercial laboratory by a positive test for rubella immunoglobulin M (IgM) from serum collected on the second day of life. Serum collected on the sixth day of life tested positive for rubella IgM and immunoglobulin G (IgG) at the Maryland State Public Health Laboratory and CDC. A throat swab collected the same day tested positive by real-time reverse transcription–polymerase chain reaction (rRT-PCR) for rubella RNA, and rubella virus genotype 1G was identified by sequencing at CDC. The viral nucleotide sequences from this CRS patient were similar to those obtained from eastern African countries bordering Tanzania. Because CRS was suspected early, appropriate specimens were collected in a timely manner, and the diagnosis was laboratory confirmed.

The mother, in her late 20s, was from urban Tanzania. She reported having a rash around the time of her first missed menstrual period in June 2011 while in Tanzania. At the time, she did not know that she was likely a few weeks pregnant. The mother’s generalized, erythematous, maculopapular rash lasted 2–3 days. She also reported swollen eyes. She reported having received all of her childhood vaccinations in Tanzania, but rubella-containing vaccine had not been part of the routine vaccination schedule. She had no prenatal care in Tanzania.

The mother arrived in the United States in December 2011, and approximately 46 days later she developed a varicella-like rash. She stated that this rash was dissimilar to the rash she had had in Tanzania. She went to a local hospital for evaluation of what appeared to be a varicella-like rash 3 days later, but laboratory testing for varicella was not performed. She went to a different hospital 13 days after rash onset with abdominal pain and concern that she had not felt the baby kick for 2 days. Fetal ultrasonography indicated breech presentation, a small abdominal circumference, and marked oligohydramnios. The next day she went to a third hospital for a prenatal visit. She still had the varicella-like rash at this visit, and it was noted that the rash was crusted over. Varicella was suspected, but testing for varicella was not performed. However, routine tests in pregnancy as described by the American College of Obstetricians and Gynecologists* include a serum test for rubella antibody, which was positive in this case (immunoglobulin type not specified) at the hospital laboratory.

### Infant B

In March 2012, an infant was born in Alabama by cesarean delivery at 33 weeks’ gestational age. At birth, the infant had generalized hemorrhagic purpura (a blueberry muffin rash) over the entire body, patent ductus arteriosus, cardiomegaly, thrombocytopenia, pneumonitis, anemia, and liver dysfunction. Approximately 1 month later, the infant was transferred to a pediatric hospital, where the infant died in April 2012. Cause of death was recorded as CRS.

Diagnosis initially was confirmed by a positive rubella IgM test result. Serum drawn from the infant on the day of birth tested positive for rubella IgM at the hospital laboratory. Serum drawn 4 days later tested positive for rubella IgM at the Alabama Bureau of Clinical Laboratories, and serum drawn 11 days after birth tested positive for rubella IgM and IgG at CDC. A throat swab and urine specimen, collected 7 days after birth, as well as a nasopharyngeal swab, collected 10 days after birth, tested positive by rRT-PCR for rubella RNA at CDC. Nucleotide sequencing identified the virus as belonging to genotype 1G and having the closest similarity to virus sequences obtained from countries neighboring Nigeria in western Africa. Because CRS was suspected at birth, appropriate specimens were collected early, enabling the diagnosis to be laboratory confirmed.

The mother was a woman in her late 20s from Nigeria. She began prenatal care in Nigeria at 9 weeks’ gestation and had a total of nine visits. Receipt of a rubella-containing vaccine, which is not part of the routine vaccination schedule in Nigeria, was not recorded at any time. She received 2 doses of tetanus toxoid and antimalarial prophylaxis at 20 and 24 weeks, respectively, but further prophylaxis was not reported. In Nigeria at 28 weeks, the baby was noted to be small for that gestational age. At 29 weeks’ gestation, the baby was noted to have asymmetric intrauterine growth retardation. No abnormal prenatal laboratory results were reported in Nigeria; however, rubella testing was not performed.

The mother arrived in the United States in early March 2012 in approximately week 32 of pregnancy. In the United States, her pregnancy was complicated with oligohydramnios and severe growth retardation. She did not recall having had a rash illness during her pregnancy. Maternal serum collected 3 days after she had given birth tested negative at CDC for rubella IgM and positive for rubella IgG with a high avidity index. Documents indicated that all members of her U.S. household (i.e., an aunt, uncle, two adolescents, and a child aged 2 years) had been vaccinated with a rubella-containing vaccine.

### Infant C

In September 2012, an infant was born in Illinois by cesarean delivery at approximately 32.5 weeks’ gestational age, weighing 1.4 lbs (650 g). Conditions noted after birth included cataracts, Dandy-Walker syndrome (discovered on antenatal ultrasound), intrauterine growth retardation, thrombocytopenia, chorioretinitis, coarctation of the aorta (which was repaired), mild liver dysfunction, mildly elevated transaminases, mild direct hyperbilirubinemia, and persistent elevation of C reactive protein. The child was discharged in February 2013.

CRS diagnosis was initially confirmed by a positive rubella IgM test result. Serum collected from the infant 44 days after birth tested positive for rubella IgM and IgG at CDC. Also at CDC, the throat swab specimen collected the same day as the serum was positive by rRT-PCR, but the nasal wash and urine specimens were negative. Nucleotide sequencing identified a genotype 1E virus, most similar to a 2011 virus from a region of Uganda bordering South Sudan and a 2008 virus from Yemen.

The mother was an immigrant from Sudan in her late 20s. Her rubella vaccination status was unknown; however, rubella vaccine is not part of the routine vaccination schedule in Sudan. The mother reported not having had a rash or fever after December 2011. She reported having had her last menstrual period in mid-January 2012. In late February, she, along with her husband and two daughters (aged 3 and 5 years), traveled by airplane to the United States via Cairo, Egypt.

The mother sought prenatal care in Illinois. Serum was drawn in early April 2012, and the rubella IgG result was positive. On the same day, the physician estimated her pregnancy at approximately 10 weeks. At the mother’s second visit, at 18 weeks, a screening test for birth defects was performed with measured levels of alpha-fetoprotein, human chorionic gonadotropin, estriol, and inhibin A; the results suggested an increased risk for Down’s syndrome. A follow-up ultrasound showed fetal abnormalities, and she was hospitalized for additional evaluation. She reported no health problems in the United States. The two daughters in the household had documented receipt of measles, mumps, and rubella vaccine, but the father’s vaccination status was unknown.

### Editorial Note

Since 2004, when rubella and CRS elimination were documented in the United States, six cases of CRS have been reported, including the three cases described here. In five cases, infection of the mother in a foreign country was thought highly probable, given travel history (i.e., Nigeria, Tanzania, Sudan, Ivory Coast, and either India, China, or Singapore). In one case, the mother did not report international travel. Although few cases of CRS have been reported in the United States, rubella continues to circulate in many other parts of the world, and the risk remains for severe effects from CRS, including death. In this report, one of the three infants with CRS died.

In 2011, a total of 130 countries, comprising approximately 41% of the world’s birth cohort, included rubella-containing vaccine in their national childhood immunization program (5). However, in the African Region, only three countries have introduced rubella-containing vaccine into their routine childhood vaccination program.† In 2009, the Region of the Americas reached its 2010 rubella and CRS elimination goal (4). The European Region and Western Pacific Region have rubella control or elimination goals, but rubella continues to circulate in these regions (6). The African, Eastern Mediterranean, and South-East Asia regions do not have a regional rubella or CRS control or elimination goal at this time because of the additional cost of the rubella component and competing priorities (e.g., polio eradication) (6).

Health-care providers should consider CRS if the mother of an infant with compatible congenital birth defects traveled during her pregnancy to an area where rubella circulates or was exposed to someone who traveled to such an area. As a nationally notifiable condition, all suspected cases of CRS should be reported immediately to the local health department, which, in turn, reports them to CDC via the state health department. Both serum and throat swab specimens should be collected as soon as CRS is suspected. Either serum positive for rubella IgM antibody or a throat swab positive for rubella RNA is confirmatory for CRS in a patient with compatible signs.

What is already known on this topic?

Congenital rubella syndrome (CRS) is caused by fetal infection with rubella virus from the mother and characterized by birth defects. During the 1964–1965 rubella epidemic in the United States, an estimated 12.5 million rubella cases occurred, and an estimated 20,000 infants were born with CRS. As a result of universal childhood vaccination, rubella and CRS elimination were documented in the United States in 2004; however, rubella still circulates in other areas of the world.

What is added by this report?

With the elimination of rubella, cases of CRS are a rare occurrence in the United States. This report describes three infants with CRS born in the United States in 2012; all had severe defects, and one died. In all three cases, the mother likely was exposed to rubella in Africa and had no documentation of vaccination against the virus.

What are the implications for public health practice?

Although CRS occurs infrequently in the United States, health-care providers and public health officials should consider CRS in an infant with compatible birth defects whose mother was in a rubella-endemic country during her pregnancy. Heightened awareness is critical for obtaining appropriate specimens early for laboratory confirmation of CRS and for initiation of a thorough epidemiologic investigation. In addition, health-care providers should know the vaccination status of women of childbearing age who are planning to travel internationally.

At this time, during maintenance of CRS elimination in the United States, confirmation at CDC of all laboratory results that support diagnoses of CRS cases is recommended. Molecular characterization of the virus is critical because the viral genotype can substantiate the suspected source of the virus or suggest one if the source is unknown, because some of the circulating genotypes are associated with specific geographic areas. Heightened awareness, gathering of pertinent information, and collection of appropriate specimens are required of the health-care provider and public health department to diagnose and investigate a case of CRS; however, these surveillance efforts are crucial to maintaining elimination in the United States.

As long as rubella remains endemic in any area of the world, imported CRS will continue to be a public health concern in the United States. Residents or foreign visitors entering the United States from rubella-endemic areas can introduce the virus. In addition, infants born with CRS can shed infectious virus for several months; therefore, care must be taken to avoid contact with others who are susceptible to rubella (e.g., unvaccinated infants in day-care settings) (7). Although levels of vaccination with rubella-containing vaccine are high in the United States, a small proportion of persons are not vaccinated for medical or personal reasons (8). Those who are not vaccinated against rubella virus can become infected if exposed. If a pregnant woman is infected with rubella virus, the fetus also can become infected. Fetal infection with rubella virus, especially early during pregnancy, often leads to CRS. The risk for CRS in the unborn child of a mother with rubella infection might be as high as 90% for infections occurring through week 10 of pregnancy (9). Clusters of unvaccinated persons are at high risk for an outbreak, as in the Netherlands and Canada in 2009 (10). Health-care providers and public health workers should remain vigilant for imported cases of CRS.

## Figures and Tables

**FIGURE:**
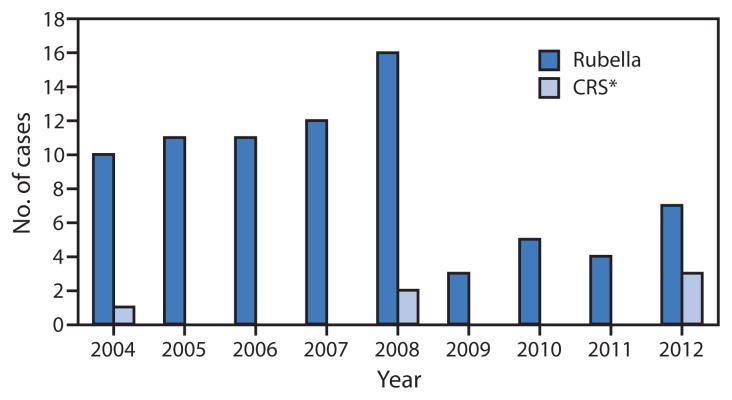
Reported cases of rubella and congenital rubella syndrome (CRS) — National Notifiable Diseases Surveillance System, United States, 2004–2012 ^*^ By year of birth.

## Footnotes

* Available at http://www.acog.org/~/media/for%20patients/faq133.pdf?dmc=1&ts=20130228t1231184484.

† Information available at http://www.who.int/entity/immunization_monitoring/data/year_vaccine_introduction.xls.
